# A Comparative Study for Assessing the Drought-Tolerance of Chickpea Under Varying Natural Growth Environments

**DOI:** 10.3389/fpls.2020.607869

**Published:** 2021-02-15

**Authors:** Anjuman Arif, Najma Parveen, Muhammad Qandeel Waheed, Rana Muhammad Atif, Irem Waqar, Tariq Mahmud Shah

**Affiliations:** ^1^Nuclear Institute of Agriculture and Biology (NIAB), Faisalabad, Pakistan; ^2^Department of Plant Breeding and Genetics, University of Agriculture Faisalabad, Faisalabad, Pakistan; ^3^Center for Advanced Studies in Agriculture and Food Security (CAS-AFS), University of Agriculture Faisalabad, Faisalabad, Pakistan

**Keywords:** chickpea (*Cicer arietinum* L.), multi-environment, genotype environment interactions, genotype selection index (GSI), drought

## Abstract

This study was planned with the purpose of evaluating the drought tolerance of advanced breeding lines of chickpea in natural field conditions. Two methods were employed to impose field conditions; the first: simulating drought stress by growing chickpea genotypes at five rainfed areas, with Faisalabad as the non-stressed control environment; and the second: planting chickpea genotypes in spring to simulate a drought stress environment, with winter-sowing serving as the non-stressed environment. Additive main effects and multiplicative interaction (AMMI) and generalized linear models (GLM) models were both found to be equally effective in extracting main effects in the rainfed experiment. Results demonstrated that environment influenced seed yield, number of primary and secondary branches, number of pods, and number of seeds most predominantly; however, genotype was the main source of variation in 100 seed weight and plant height. The GGE biplot showed that Faisalabad, Kallur Kot, and Bhakkar were contributing the most in the GEI, respectively, while Bahawalpur, Bhawana, and Karor were relatively stable environments, respectively. Faisalabad was the most, and Bhakkar the least productive in terms of seed yield. The best genotypes to grow in non-stressed environments were CH39/08, CH40/09, and CH15/11, whereas CH28/07 and CH39/08 were found suitable for both conditions. CH55/09 displayed the best performance in stress conditions only. The AMMI stability and drought-tolerance indices enabled us to select genotypes with differential performance in both conditions. It is therefore concluded that the spring-sown experiment revealed a high-grade drought stress imposition on plants, and that the genotypes selected by both methods shared quite similar rankings, and also that manually computed drought-tolerance indices are also comparable for usage for better genotypic selections. This study could provide sufficient evidence for using the aforementioned as drought-tolerance evaluation methods, especially for countries and research organizations who have limited resources and funding for conducting multilocation trials, and performing sophisticated analyses on expensive software.

## Introduction

Drought is accepted as a serious threat to crops worldwide, more specifically to the areas where there is less rain fall. Grain yield is considered to be a determining factor of stress tolerance in crop plants as scarcity of water leads to reduced grain yield in cereal and legume crops. This has been indicated in a meta-analysis of more than 100 studies where chickpea stands on the seventh position among 13 legume species when categorized on the basis of drought-induced yield reduction (Daryanto et al., 2015).

Chickpea is the most important legume crop and a source of nutrition to millions of people globally due to its richness in protein, fiber, and minerals (Dadon Bar-El et al., 2017). It also re-mediates the soil by its ability to fix nitrogen in a symbiotic relationship with rhizobacteria upon nodulation. Pakistan is the third largest producer of chickpea in the world (FAOSTAT, 2015), and among all provinces, Punjab contributes 80% of chickpea production in Pakistan (Government of Pakistan [GOP], 2016). It is mainly cultivated in marginal lands, rainfed areas, and in sand dunes of the Thal desert (Rafiq et al., 2018; Nisa et al., 2020).

Besides, chickpea is a cool season crop and grown mainly in rainfed areas where it suffers terminal drought at the time of maturity and seed filling due to increase in temperature and reduced or no rainfall (if occurs), which causes drastic yield losses. Planting in spring rather than winter also exposed chickpea plants to face drought stress (Toker and Cagirgan, 1998; Richards et al., 2020).

High yielding genotypes when grown under limited water availability were used to be selected as drought tolerant varieties. Due to variations in weather conditions observed each coming year leads to variation in the pattern of onset of drought stress. Hence, selection on the basis of grain yield only seems not sufficient to produce high yielding drought tolerant varieties in the climate change scenario (Ludlow and Muchow, 1990; Blum, 1996).

Chickpea yield varies and is strongly influenced by environmental factors, i.e., photoperiod, temperature (Velu and Shunmugavalli, 2005), and it depends upon differential composition of soil type and available water, changing weather conditions on the same location (environment) in different years, or in multiple locations in one season or over years (Crossa, 1990; Purchase et al., 2000). This can be explained by genotype and environment (GE) interaction, which described that two genotypes in the same environment behave differently due to variations in their genetic makeup. Interactions can also be distinguished on the basis of whether they are repeatable or non-repeatable within the target genotype–environment system. Similarly, a specific environment can influence one genotype in a different way than other genotypes, or a single genotype behaves differently in different environments (Falconer, 1981; Misra et al., 2020; Nisa et al., 2020). However, genotypes with wider adaptability to a number of diverse environments without affecting their yield potential seems to be a better strategy, which needs extensive multi-location trials of genotypes under study consecutively for 2–3 years (Farshadfar et al., 2011).

The additive main effects and multiplicative interaction (AMMI) model has been used extensively in agriculture research for the evaluation of breeding lines prior to varietal approval. AMMI first applies analysis of variance (ANOVA) to partition the variation into genotype main effects (G), environment main effects (E), and genotype-by-environment interaction effects GE), and then it applies principal components analysis (PCA) by singular value decomposition to GE (Gauch, 1992, 2006; Yan et al., 2007; Gauch et al., 2008; Yang et al., 2009).

Generalized linear models (GLM) also used to measure variations contributed by genotypes, environment, and G × E interactions for each response variable by combined analysis of variance (ANOVA) and linear regression. GLMs relax the assumption about the dependent variables (Olsson, 2002).

The ability of crop cultivars to perform reasonably well in drought-stressed environments is paramount, and non-stressed environments can be used as an indicator to identify drought-resistant varieties in breeding for drought-prone environments. Several drought indices have been suggested on the basis of a mathematical relationship between yield under drought conditions and non-stressed conditions. These indices are based on either drought resistance or drought susceptibility of genotypes (Clarke et al., 1984).

In the context of ongoing international research on G × E interactions and the development of sustainable climate-resilient drought-tolerant chickpea genotypes, we realized the need for a large-scale cohesive study to evaluate the advanced breeding lines for drought tolerance based on the stability and yield prior to being subjected to the National Uniform Yield Trials, a pre-requisite of variety approval. We hypothesized that the breeders’ lines coming from all over Pakistan from Government Agriculture Research Organizations for preliminary yield trials would be a good start. We planned the study keeping this in mind.

The objectives of this study were the assessment of yield potential, stability, and drought tolerance of chickpea genotypes under natural field conditions, and to identify discriminating environment-specific genotypes, and genotypes have wider adaptability for a range of environments.

## Materials and Methods

The germplasm used in the study were advanced breeding lines of chickpea. A set of 83 advanced breeding lines with commercial varieties as checks were acquired from the provincial and national uniform yield trials conducted at multiple locations across Pakistan (Supplementary Table 1) in the season of 2016–2017. In the next year (2017–2018), 80 genotypes were evaluated for drought tolerance, 50 from the previous year’s trials and the rest were advanced breeding lines acquired from the Nuclear Institute for Agriculture and Biology (NIAB), Pakistan. In the third year of the study, 40 genotypes were selected from 80 genotypes tested in 2017–2018 on the basis of yield performance.

### Experimental Design

The trials were laid out according to the randomized complete block design (RCBD), replicated four times with a plot size of 1.8 m^2^ giving a plant density of approximately 25 plants per m^2^. The harvesting and threshing were conducted manually.

### Imposition of Field Drought Stress and Data Recording

The study was planned to evaluate the drought tolerance of advanced breeding lines of chickpea in natural field conditions. Two methods were employed to impose drought-stressed field conditions; Experiment 1, by growing chickpea genotypes at five locations in rainfed chickpea-growing areas (four of which were situated in the Thal desert of Pakistan) with only natural rainfall and no supplemental irrigation, and at an irrigated location, i.e., Faisalabad City of Pakistan, as the non-stressed environment. Descriptions of the planting environments are described in Table 1. Data were recorded, for observations, i.e., *Seed yield* (SY), *hundred-seed-weight* (100SW), *number of seeds* (NOS), *number of pods* (NOP), *number of primary branches* (NPB), *number of secondary branches* (NSB), and *plant height* (PH). Drought tolerance score (DRS) was estimated using a rating scale ranging from 1 (plants with 95–100% pod setting) to 9 (plants did not set any pods and dried out).

**TABLE 1:** Environment types, soil properties, and geographic coordinates of environments.

**Sr. #**	**Name**	**Type**	**Soil Type**	**Latitude, Longitude, Sea level**
1	Karor	Rainfed	Calcareous, Sandy loam (light)	31.2301, 70.9475, 155 m
2	Bhakkar	Rainfed	Silt loam, Silty clay loam, Clay loam	31.6303, 71.0676, 169 m
3	Kallur Kot	Rainfed	Sandy loam	32.1567, 71.2724, 191 m
4	Bahawalpur	Rainfed	Silt loam, Silty clay loam, Clay loam	29.3946, 71.6638, 116 m
5	Faisalabad	Rainfed, Irrigated	Silt loam or very fine sandy loam	31.4126, 3.0551, 184 m
6	Bhawana	Rainfed	Silt loam or very fine sandy loam	31.5685, 72.6485, 172 m

*Calcareous and clayey soils: potential productivity is high with adequate water and nutrients supply. Sandy loams: are capable of quickly draining excess water and usually deficient in micronutrients, especially zinc and iron. Silt soils: are fertile, light but moisture-retentive and easily compacted.*

Experiment 2 involved the planting of chickpea genotypes in spring as simulating a drought stressed environment (S2), whereas winter-sowing was regarded as the non-stressed environment (S1). Five observations, i.e., *seed yield* (SY), *biological yield* (BY), *seed weight* (SW), *harvest index* (HI), and *plant height* (PH) were recorded under stressed and non-stressed conditions. The biological yield was recorded as shoot dry-weight (g) per plant, and the harvest index was calculated as described by El Naim et al. (2010). The formula is given below:

$$H\,a\,r\,v\,e\,s\,t\,I\,n\,d\,e\,x=\frac{\mathrm{Seeds}\,\mathrm{yield}\,\left(\text{g}\right)}{\mathrm{Shoot}\,\mathrm{dry}\,\mathrm{weight}\,\left(\text{g}\right)}$$

Weather data was collected from *https://www.worldweatheronline.com/*. Soil texture and physical properties account for a major proportion in environmental characterization, and therefore, the soil types of six environments are listed in Table 1.

### G × E Data Analyses

#### AMMI and GLM Models

Generally, genotype–environment interaction (GEI) is common when genotypes (G) are tested across a number of environments (E). The GE main effects were extracted using two statistical models, i.e., the AMMI and GLM.

The AMMI analysis has two parts. First, the analysis of variance of genotype (G), environment (E), and genotype–environment (GE) interactions (subdivided into principal components called interaction principal components or IPCAs) and AMMI main effect biplot in which GE means are plotted on the *x*-axis, while the IPCA1 scores are on the *y*-axis. Second is the genotype (G) and genotype–environment interaction (GEI) biplot (Yan and Tinker, 2006). There are several types of biplot options available in GenStat to plot GEI. On the other hand, GLM is basically analysis of variance and generalized linear regression without any principal component analysis. GenStat software was used for the AMMI and GGE biplot analysis, whereas MINITAB 14 was used for GLM analysis.

#### Genotype Selection Index

The AMMI stability value (ASV) was manually computed as per Purchase (2000); however, the IPCA sum of squares of interactions and IPCA1 and IPCA2 scores were used from the AMMI analysis using the GenStat software. The equation was as follows:

AMMI⁢Stability⁢Value⁢(ASV)=

$$\sqrt{{\left[\frac{\text{IPCA}\,1\,\mathrm{Sum}\,\mathrm{of}\,\mathrm{Squares}}{\text{IPCA}\,2\,\mathrm{Sum}\,\mathrm{of}\,\mathrm{Squares}}\,\left(\text{IPCA}\,1\,\text{score}\right)\right]}^{2}+{\left[\text{IPCA}\,2\,\mathrm{score}\right]}^{2}}$$

When computing ASV using Microsoft Excel, the following formula was used:

**SQRT[(IPCA1 sum of squares/IPCA2 sum of squares^∗^IPCA1 score)^2 + (IPCA2^2)]**.

The AMMI stability value (ASV) and the mean seed yield of individual genotypes across environments were used to derive another component of stability and yield-based selection of genotypes across environments as genomic selection index (GSI), described by Farshadfar (2008) and Pouresmael et al. (2018). We have modified its name to be “genotype selection index” rather than “genomic selection index.” The genotype selection index (GSI) was calculated using the following formula:

GSI=RASV+RY

where RASV and RY are the rank of the AMMI stability value and the mean seed yield of a genotype, respectively. Ranking was performed with the lowest AMMI value and the highest mean seed yield ranked one. Lower ASV values indicate greater stability for a genotype; conversely, the less stable genotypes are those with close to maximum amounts of GSI. Therefore, GSI combines both mean yield and stability in a single criterion.

### Drought Tolerance Indices

The mean yield of each genotype was recorded in stressed (Ys) and non-stressed (Yp) conditions and used to calculate eight drought stress tolerance indices to assess yield- and stability-based selections of genotypes under simulated stressed and non-stressed natural field conditions. Formulas are presented in Table 2. According to a classification by Fernandez (1992), he distributed the genotypes evaluated by drought tolerance indices into Group A consisting of genotypes with high seed yield under both drought stress and non-stressed conditions, Group B, comprised of genotypes with high yield only under non-stress conditions, Group C, genotypes could produce good yield only under stress conditions, whereas genotypes with poor yield performance under both conditions were categorized as Group D.

**TABLE 2:** Drought tolerance indices, formulas, and references.

**Index**	**Formula**	**References**
*Stress Tolerance*	TOL = Yp–Ys	Rosielle and Hamblin (1981)
*Mean Productivity*	MP = (Yp + Ys)/2	Rosielle and Hamblin (1981)
*Geometric Mean Productivity*	GMP = (Yp * Ys) ^0^.^5^	Fernandez (1992)
*Stress Susceptibility Index*	SSI = [(1–(Ys/Yp)]/SI	Fischer and Maurer (1978)
*Stress Index*	SI = 1–(Ỹs/Ỹp)	Fischer and Maurer (1978)
*Stress Tolerance Index*	STI = (Yp * Ys)/(Ỹp) ^2^	Fernandez (1992)
*Yield Index*	YI = Ys/Ỹs	Gavuzzi et al. (1997)
*Yield Stability Index*	YSI = Ys/Yp	Bouslama and Schapaugh (1984)

*Yp and Ys, seed yield of each genotype under non-stress and stress conditions, respectively. Ỹp and Ỹs, mean seed yield of all genotypes under non-stress and stress conditions, respectively.*

### Graphics

Trellis plots and G × E plots were produced using GenStat software. R package “Corr,” “Corrplot,” and “Corrgram” were used for computing correlation matrix and graphics. However ggplot2 was used to produce boxplots.

## Results

Environmental and genetic factors impact certain metabolic processes that influence growth and yield in a crop, and quantitatively understanding the correlations between these factors and phenological development can help us predict crop yield. Emergence, flowering, pod set, and physiological maturity are defined as the four developmental stages of chickpea (*Cicer arietinum* L.). The last three stages are indeterminate and occur concurrently in different parts of the plant along with the vegetative growth (Summerfield and Wien, 1980; Saxena, 1984). Temperature, photoperiod, and available moisture are generally most influential to chickpea development (Soltani et al., 1999).

### Evaluation of Drought Tolerance Under Naturally Stressed and Non-stressed Environments

In this experiment, diverse chickpea genotypes were characterized for their drought stress tolerance on the basis of phenotypic traits through replicated yield trials (2017–2018) in five rainfed areas, which are referred to as *stressed environments*. Conversely, E5 was considered a non-stressed environment due to its soil type and irrigation system (Table 1).

**FIGURE 1:**
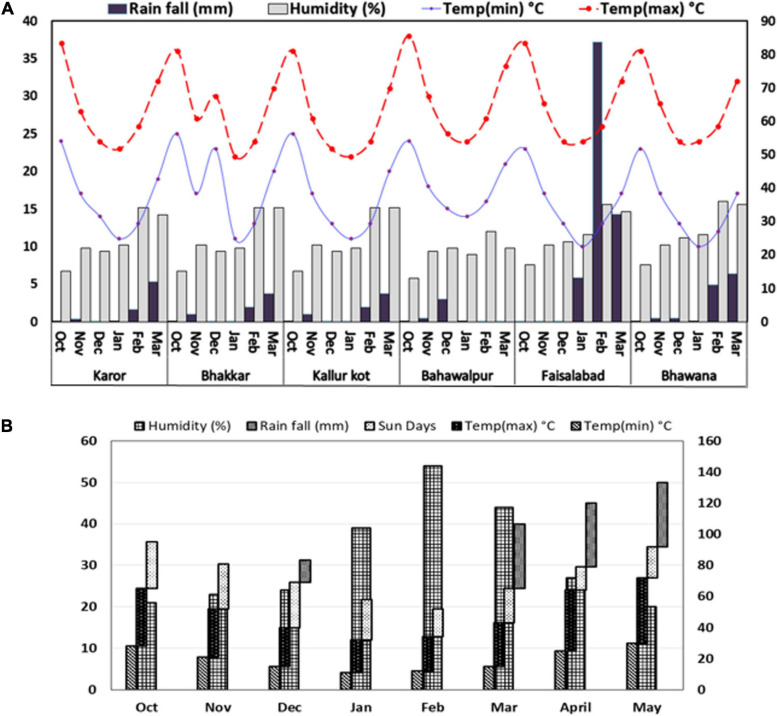
Weather footprint describing monthly rainfall (mm), relative humidity (*%*), and extreme maximum and minimum temperatures °C in growth period of Chickpea genotypes grown under natural **(A)** drought stressed and non-stressed and **(B)** simulated drought stressed and non-stressed field conditions.

The weather data of drought stressed environments were almost similar: dry with low rainfall, i.e., 16 mm at E3 and E2, 17 mm at E1, 8 mm at E4, and 27 mm at E6. The non-stressed environment E5, on the contrary, was very wet with 129 mm rainfall during the crop period (Figure 1A). The weather data recorded for Experiment 2 are shown in Figure 1B. The temperature from January to February was favorable for the chickpea plant, but these months were quite dry, as no rainfall was recorded after the spring sowing. Later, day and night temperatures rose in March and April, but these months had 82 mm rainfall, and decreased humidity due to the rise in temperature.

The average temperature at four environments was similar in these months but a rise in temperature of 1–2°C was noted in E1, and E4 in the months of October, February, and March. E4 had the highest average temperature among all six locations. The rainfall received was the least in E4 and the greatest in E5 (Figure 1A). It is suggested that E5 was found the most favorable and E4 with the most adverse soil characteristics among all six locations.

#### Estimation of Phenotypic Variations Among Environments

**FIGURE 2:**
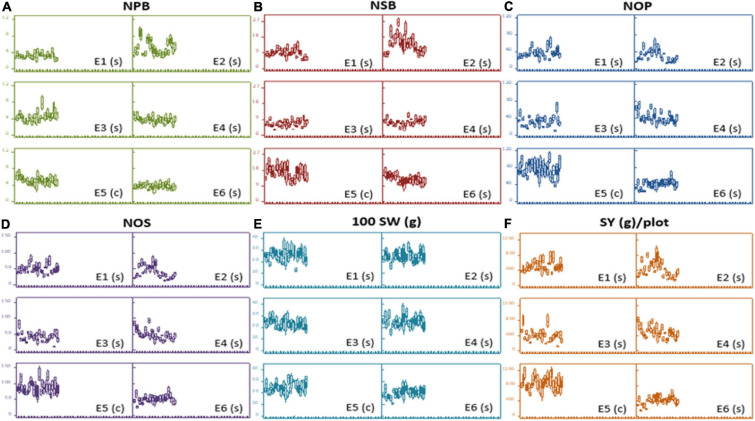
Trellis plots exhibiting distribution of phenotypic data of different agronomic traits: **(A)** Number of primary branches (NPB). **(B)** Number of secondary branches (NSB). **(C)** number of pods (NOP), **(D)** number of seeds (NOS), **(E)** hundred-seed-weight [100SW(g)], and **(F)** seed yield [SY(g)/plot across six varying environments]. Each boxplot represents a data subset of genotypes with similar values in the data set instead of blurring the effect of each other in a single boxplot of each genotype.

**FIGURE 3:**
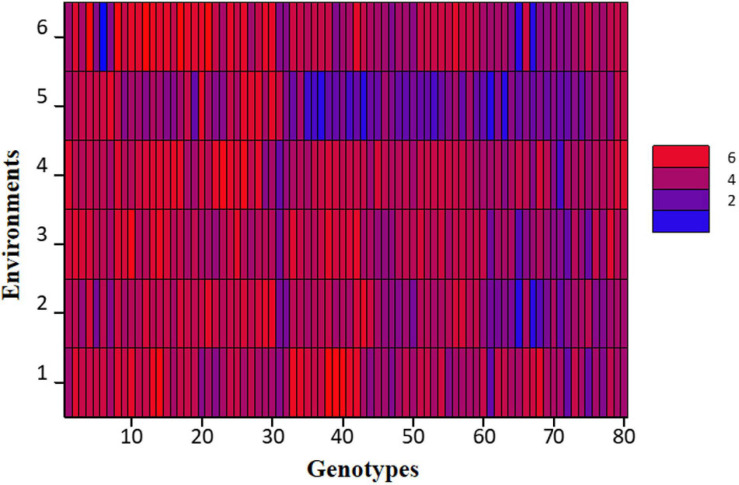
Shade plot of drought tolerance scores of 80 chickpea genotypes across six environments.

At large, trellis plots of environments described that environments have profound effects on individual observation. Environment influences GY, NPB, NSB, NOP, and NOS in the most predominant way; however, genotype was the main source of variation in 100 SW and PH. Genotype means of NPB and NSB was highest in Bhakkar, but Faisalabad was best for genotypes to attain maximum PH, NOP, NOS, and SY (Figure 2). DRS exhibited that the intensity of drought was minimum in Faisalabad, i.e., the non-stressed environment (Figure 3).

#### Correlation of Phenotypic Variations Among Environments

**FIGURE 4:**
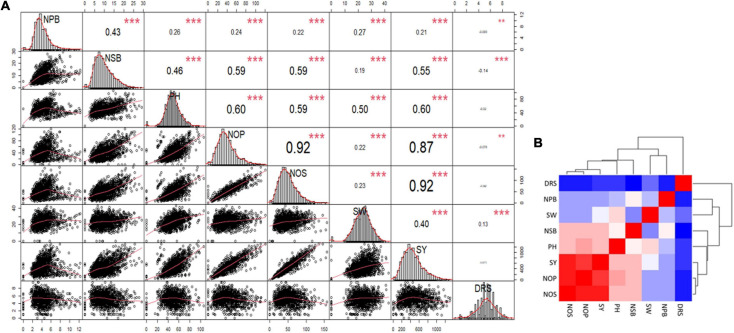
**(A)** Correlation Matrix of the data for number of primary branches (NPB), number of secondary branches (NSB), plant height (PH), number of pods (NOP), number of seeds (NOS), hundred-seed-weight (SW), seed yield (SY), and Drought Tolerance Score (DRS). The distribution of each variable is shown on the diagonal. On the bottom of the diagonal: the bivariate scatter plots with a fitted line are displayed. On the top of the diagonal: the value of the correlation plus the significance level as stars. Color intensity and the size of the correlation values are proportional to the correlation coefficients. **(B)** Heat map and clustering of the correlation matrix, red color shows maximum correlation followed by pink then white and blue shows minimum or no correlation among the traits.

A correlation matrix of the mean data for NPB and NSB, PH, NOP, NOS, and SY showed a high positive correlation in NOP, NOS, and GY, and a strong positive correlation in PH, NOP, NOS, and GY. Increase in NSB also correlated with increases in NOP, NOS, and SY. NPB displayed a relatively low level of positive correlation with PH, NOP, NOS, 100 SW, and SY. NPB and NSB had moderately strong correlation. Drought score exhibited high negative correlation with SY and significant negative correlation with PH and NOS, whereas it showed no correlation with other traits (Figure 4A). A heat map provided a visual overview, and relative intensity of correlation values in a pair of observations demonstrated the extent and dimension of correlation. For instance, NOS, NOP, and SY shared a red colored grid of nine squares describing a strong positive correlation among them (Figure 4B).

#### Estimation of Genotypic Variations Among Environments

**FIGURE 5:**
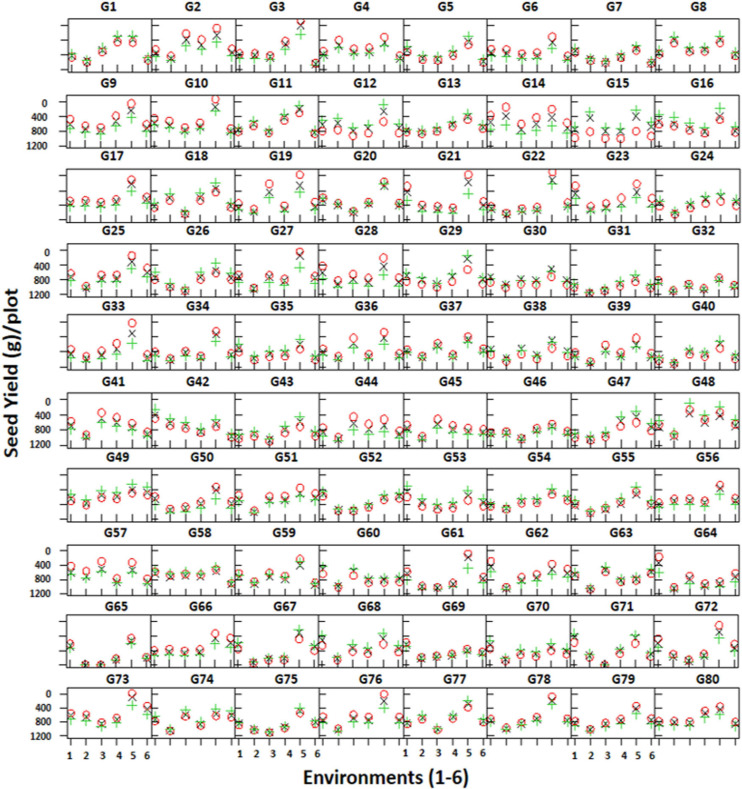
Trellis plots showing mean seed yield (g/plot) of 80 genotypes across six environments. Genotypes are coded as G1–G80.

Trellis plots showing mean seed yield of individual genotypes clearly demonstrate that seed yield was higher in the non-stressed environment (E5) compared to the stressed environments (Figure 5). The trellis plots have shown that genotypes CH28/07 (G48) and CH10/08 (G49) performed well in most of the environments; however, DCD (G3) produced the highest mean seed yield at the non-stressed environment (E5). Similarly, some genotypes like CM616/10 (G41), TG12K10 (G45), and CH23/00 (G64) produced better mean seed yield in stressed environments.

#### Genotype × Environment Interaction

##### Genotype main effects: additive main effects and multiplicative interaction biplot

**FIGURE 6:**
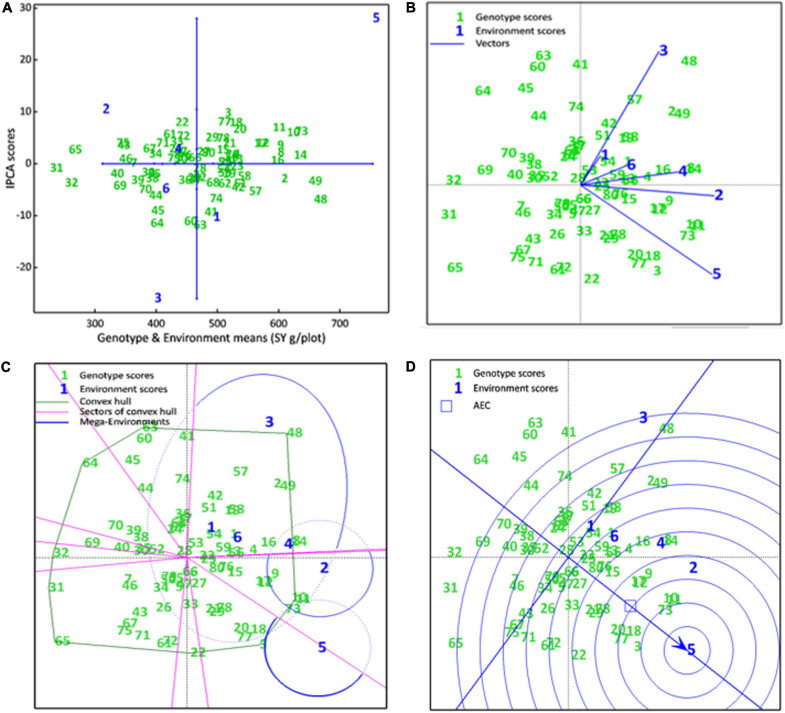
**(A)** AMMI main effects biplot. SY of genotypes are plotted on the *x*-axis, while the IPCA1 scores are on the *y*-axis. The origin represents the average performance of all genotypes in each environment and there is zero GEL. Displacement along the *x*-axis shows differences in the main effects, whereas displacement along the *y*-axis reflects differences in the interaction effects. **(B)** The scatter plot of seed yield data of chickpea genotypes across six environments explained 58.52% of total variations. Axis 1 explains IPC1 (35.71%) along *x*-axis, axis 2 explains IPC2 (22.81%). **(C)** Which-won-where view of the biplot. A convex hull polygon around all genotypes scored has been drawn by connecting the farthest genotypes to form a polygon that encompasses all genotypes. Sectors draw lines from the origin perpendicular to each side of convex hull, that divide the biplot into sectors, while mega environments draw an ellipse around the environments that share the same sectors. **(D)** A Comparison biplot. Concentric circles plotted around ideal environment, the smaller the circle containing an environment, the more attributes it shares with ideal environment. The smaller circle containing a genotype, the greater is its stability and performance in an ideal environment

The AMMI main effects biplot helps to isolate the genotypes producing high yields and broadly (near the origin) or specifically (far from origin) adapted to nearby environments in biplot (Figure 6A). Genotypes like CH76/08 (G28), CH2/11 (G52), Bittle-2016 (G66), and AZC (G6) were plotted near the origin. This indicates that these were less sensitive to environmental interaction and broadly adapted with near average SY. However, G14 (D-13029), G16 (CM584/09), D-13036 (G8), D-13012 (G9), CH 39/08 (G2), CH28/07 (G48), and CH10/08 (G49) had a better mean seed yield than the mean of all genotypes and plotted far from the origin along the *x*-axis but close to the origin along the *y*-axis. These were suggested high-yielding and relatively insensitive to GEI. Similarly, CM877/10 (G40) and TG12K02 (G46) performed less than the grand mean yield, and plotted far from the origin on the x-axis but nearer to the *y*-axis, meaning that these were low-yielding genotypes and insensitive to GEI. DG-2017 (G31) and QG-1 (G32) genotypes belong to Province Sindh and were not able to adapt in Punjab. Moreover, ILC3279 (G65), a traditional cultivar acquired as germplasm from ICRISAT, produced very poor SY in comparison with the grand mean. Similarly, environments near the origin along the *y*-axis with a lesser IPCA score contributed little in GEI, such as E4 and E6, while E1 and E2 elicited a moderate level of GEI, but E3 elicited strong interactive forces, and above all, E5 contributed most in terms of GEI as it was spotted farthest point on biplot. E5 also plotted from the origin along the *x*-axis and was the most productive environment in terms of producing SY, followed by E1, which had mean yield little better than average means. All other environments produced seed yields less than average where E2 was the least productive among all. Ranking of all genotypes according to AMMI estimates in all environments is presented in Supplementary Table 2. The first four AMMI selections in each environment and a hierarchical cluster analysis clustered genotypes according to the AMMI estimates (Table 3).

**TABLE 3:** First four additive main effects and multiplicative interaction (AMMI) selections per environment with mean yield, variance, and score.

**Environment**	**Mean**	**Variance**	**Score**	**1**	**2**	**3**	**4**
E5	753.7	48,780	27.99	DCD (G3)	BRC-457 (G11)	CH15/11 (G73)	D-14005 (G10)
E2	312.5	34,556	10.45	CH15/11 (G73)	D-14005 (G10	D-13029 (14)	BRC-457 (G11)
E4	430.6	23,617	2.77	CH28/07 (G48)	CH39/08 (2)	CH10/08 (49)	BRC-457 (G11)
E6	410.2	19,273	-4.89	CH10/08 (49)	D-13029 (14)	CH28/07 (G48)	CH15/11 (G73)
E1	493.4	25,420	-10.34	CH23/00 (64)	*K-01219* (42)	CH3/11 (53)	CH2/11 (52)
E3	397.1	37,473	-25.99	CH28/07 (G48)	CH39/08 (2)	*CM616/10* (41)	CH10/08 (49)

##### Genotype main effects: analysis of variance (additive main effects and multiplicative interaction and generalized linear model)

As per good practice, GLM was applied to our data in addition to AMMI to compare analytical competitiveness of GLM with special software-based AMMI analysis. An analysis of variance revealed that both models produced the same results (Table 4). Both models revealed that the genetic makeup of genotypes contributed least in the phenotypic variations of all traits in comparison to the environment and GEI. However, it did have a significant influence when SW and DRS were taken into account. NSB, NOP, NOS, and SY were most influenced by environmental factors, whereas NPB, PH, 100 SW, and DRS were most affected by GEI. The number of secondary branches (NSB) produced were greatly influenced by the environment alone. Environment means and variances of SY showed that the non-stressed environment, i.e., Faisalabad elicited the highest interactive forces on genotypes than the stressed environments.

**TABLE 4:** Additive main effects and multiplicative interaction (AMMI) and generalized linear regression model (GLM) analysis of variance of the 80 chickpea genotypes tested across six environments.

**Source**	**Number of primary branches**				**Number of secondary branches**				**Plant height (cm)**				**Number of pods**			
				
	**GLM**	**Var.**	**AMMI**	**Var.**	**GLM**	**Var.**	**AMMI**	**Var.**	**GLM**	**Var.**	**AMMI**	**Var.**	**GLM**	**Var.**	**AMMI**	**Var.**
	
	**SS**	**%**	**SS**	**%**	**SS**	**%**	**SS**	**%**	**SS**	**%**	**SS**	**%**	**SS**	**%**	**SS**	**%**
Genotype (G)	464	12	468	12	2,832	9	2,878	9	33,961	13	33,901	13	51,230	10	50,959	10
Environment (E)	872	23	873	23	15,323	50	15,404	50	49,882	20	50,947	20	232,051	44	234,735	44
G × E	1,851	49	1,864	49	8,478	28	8,602	28	90,199	35	92,578	36	182,482	35	183,380	35
*IPCA1*	–	–	*1,123*	*29*	–	–	*3,948*	*13*	–	–	*37,984*	*15*	–	–	*67,113*	*13*
*IPCA2*	–	–	*400*	*10*	–	–	*1,709*	*6*	–	–	*27,496*	*11*	–	–	*43,912*	*8*
*Residuals*	–	–	*341*	*9*	–	–	*2,945*	*10*	–	–	*27,098*	*10*	–	–	*72,356*	*14*
Error	618	16	604	16	3,728	12	3,644	12	80,869	32	79,053	31	61,621	12	59,918	11
Total	3,804	100	3,822	100	30,362	100	30,612	100	25,4912	100	258,310	100	527,383	100	530,733	100
*R*-squared (%)	0.84	–	–	–	0.88	–	–	–	0.68	–	–	–	0.88	–	–	–
	
**Source**	**Number of seeds**				**100 grain weight (g)**				**Grain yield (g)**				**Drought Score**			
	
	GLM	Var.	AMMI	Var.	GLM	Var.	AMMI	Var.	GLM	Var.	AMMI	Var.	GLM	Var.	AMMI	Var.
	SS	%	SS	%	SS	%	SS	%	SS	%	SS	%	SS	%	SS	%
Genotype (G)	107,060	14	107,232	14	11,796	22	11,994	23	7,238	16	7,267	16	877	26	882	29
Environment (E)	313,954	40	315,338	40	4,036	8	4,067	8	17,309	38	17,432	38	368	11	399	13
G × E	275,354	35	276,180	35	18,383	35	18,795	35	15,620	34	15,689	34	1,334	39	1,259	41
*IPCA1*	–	–	*99,119*	*13*	–	–	*6,804*	*13*	–	–	*5,463*	*12*	–	–	*504*	*16*
*IPCA2*	–	–	*58,093*	*7*	–	–	*4,199*	*8*	–	–	*3,765*	*8*	–	–	*440*	*14*
*Residuals*	–	–	*118,968*	*15*	–	–	*7,792*	*15*	–	–	*6,461*	*14*	–	–	*316*	*10*
Error	92,680	12	90,561	11	18,284	35	17,880	34	5,291	12	5,153	11	853	25	539	17
Total	789,048	100	791,481	100	52,498	100	53,147	100	45,457	100	45,681	100	3,431	100	3,079	100
*R*-squared (%)	0.88	–	–	–	0.65	–	–	–	0.88	–	–	–	0.75	–	–	–

*Var, variations; NPB, number of primary branches; NSB, number of secondary branches; PH, plant height; NOP, number of pods; NOS, number of seeds; SW, hundred-seed-weight; SY, seed yield; and DRS, drought tolerance score.*

Genotype by environment interactions (GEIs) affect how well a genotype performs in different environments. The AMMI GGE biplot, which is statistically a scatter plot, is summarized by the two interactive principal component (IPCA) axes. Axis 1 explains IPC1, axis 2 explains IPC2 and its origin represents no GEI. The scatter plot of seed yield data showed a positive correlation between all environments as indicated by an acute angle between them (Figure 6B). Genotypes D-14005 (G10), BRC-457 (G11), and CH15/11 (G73) clustered together, showing similar seed yields across environments and being influenced by GEI in a similar way. However, environments E4, E6, and E1 tended to cluster together influencing the genotypes in a similar way. Environments E4 and E6 close to the origin elicited weak interactive forces, whereas E5 was far from the origin and was suggested to elicit strong interactive forces. Genotypes CH28/07 (G48) and D-07509 (G63) were plotted far from the origin, and so were more prone to being influenced by GEI. G48 was plotted near E3 and was hence specifically adapted to E3, similarly D-14005 (G10), BRC-457 (G11), CH15/11 (G73), G18 (K-01216), and CH55/09 (G20) were specifically adapted and positively correlated to the non-stressed environment, i.e., E5.

#### Selection of Best Suitable Genotype and Environment

GGE plots can be improved by generating different types of biplots. Some are used here for creating better visuals of G × E analyses.

##### Which-won-where

A which-won-where view of the biplot helps to identify which genotypes performed best in each environment and in each mega environment. Our results revealed that the seed yield biplot contained three mega environments (ME): ME1, containing E1, E2, E3 (E3), E4, and E6; ME2, containing E2 and E4; and third; ME3 consisting of E5 only, which is a non-stressed and ideal environment as well (Figure 6C). As a general rule, genotypes that appear in the same sectors as a particular environment are the best performers in that environment, e.g., genotypes D-13012 (G9), D-14005 (G10), BRC-457 (G11), D-13029 (G14), and CH15/11 (G73) were located in the ME2 and are expected to produce maximum seed yield in these environments, whereas E5 was the most suitable for DCD (G3) and CH15/11 (G73) for producing maximum seed yield.

##### Comparison biplot

A comparison biplot is used to compare the performance of an environment with that of an ideal environment. In a comparison, E4 shares more attributes with E5 than with E2, E3 (E3), E6, and E1 (in decreasing order of common attributes). In addition, the smaller the circle containing a genotype, the greater is its stability and performance in an ideal environment. Genotypes DCD (G3), CH15/11 (G73), D-14005 (G10), BRC-457 (G11), K-01216 (G18), and CH55/09 (G20) are likely to be ideal genotypes in the ideal environment E5 in terms of achieving higher mean seed yield, good stability showing a low IPCA score (Figure 6D).

##### Ranking biplot

**FIGURE 7:**
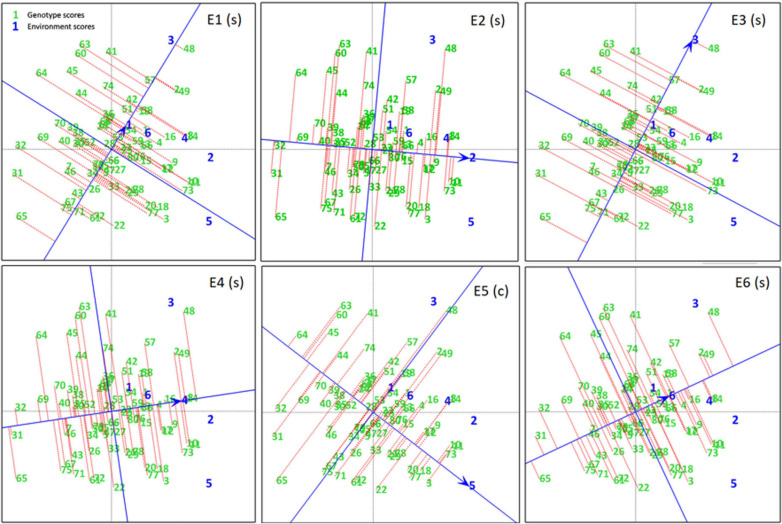
Experiment 1: Ranking Plots of seed yield data of chickpea genotypes across six environments **(E1–E6)**. Arrows on the axis point in the direction of higher means performance of the genotype and consequently rank the genotypes with respect to their performance. Lines projected from the genotypes onto the axes to see that genotypes are ranked in descending order, the length of the line is a measure of genotype stability. Short lines indicate high stability, while long lines indicate low stability.

A ranking plot is a presentable method for showing the best performing genotypes in a specific environment and can also be used to show the best environment for a specific genotype. Genotypes DCD (G3), CH15/11 (G73), D-14005 (G10), BRC-457 (G11), K-01216 (G18), and so on are the best-performing genotypes in terms of seed yield in E5. Similarly Figure 7 shows the ranking of all genotypes in each environment (A–E) on the basis of their yield performance.

#### Genotypic Selections by Additive Main Effects and Multiplicative Interaction Model Based Indices

We found that on the basis of ASV, genotypes, for example, DG-2017 (G31), TG12K-07 (G25), CH76/08 (G28), and AZC (G6) were the top-most stable genotypes; however, based on yield performances, these were on the 80th, 28th, 44th, and 48th place in rankings (Supplementary Table 3). We identified that the GSI ranking is comparable to the AMMI model-based ranking (Supplementary Tables 2, 3). Both identified DCD (G3), CH15/11 (G73), D-14005 (G10), BRC-457 (G11), K-01216 (G18), CH55/09 (G20), and CH28/07 (G48) as the few top-most stable and high-yielding genotypes in all environments. Similarly, both methods ranked the same genotypes, e.g., TG12K-07 (G25), K-01250 (G39), CM877/10 (G40), CH76/08 (G28), K-01302 (G38), K-01240 (G43), K-01308 (G34), K-01242 (G35), and K-01209 (G30) as low yielding in comparison to mean yield and relatively less stable in the list.

### Evaluation of Drought Tolerance in Simulating Stressed and Non-stressed Environments

**FIGURE 8:**
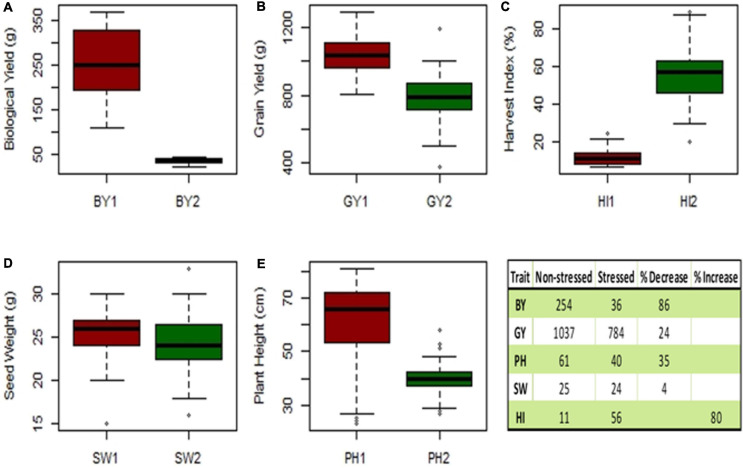
Experiment 2; Box plot of mean data traits of **(A)** *biological yield* (BY), **(B)** *seed yield* (SY), **(C)** *harvest index* (HI), **(D)** *seed weight* (SW), and **(E)** *plant height* (PH). Table has shown the reduction (%) observed in the mean of non-stressed (1) vs. drought-stressed genotypes (2).

A box plot was used to differentiate the distinct values of mean data for observations, i.e., *seed yield* (SY), *biological yield* (BY), *seed weight* (SW), *harvest index* (HI), and *plant height* (PH) were recorded for 40 genotypes and quantified to assess their performance under the winter and spring sowings. The reduction observed in the mean GY, BY, SW, and PH of drought-stressed or spring-sown chickpea genotypes in comparison to those that were non-stressed or winter-sown was 24, 86, 4, and 34%, respectively. On the contrary, HI increased by 80% in the stressed conditions due to less vegetative and more reproductive growth (Figure 8).

#### Analysis of Variance

Analysis of variance revealed that seed yield, biological yield, and plant height were more influenced by genotypic effects than by environment, and GEI indicates the germplasm tested was genetically diverse (Table 5). However, most of the variation in SY (58%) remained unexplained. The harvest index was mainly influenced by environment. Total variations measured in seed weight in two environments were 98% attributed to E and GEI (49% each).

**TABLE 5:** Analysis of variance (ANOVA) for biological yield (BY), seed yield (SY), harvest index (HI), seed weight (SW), and plant height (PH).

**Source**	**Grain Yield**		**Biological Yield**		**Harvest Index**		**Seed Weight**		**Plant Height**	
	**SS**	**Var.%**	**SS**	**Var.%**	**SS**	**Var.%**	**SS**	**Var.%**	**SS**	**Var.%**
Genotype (G)	3,504.64	18	4,660,202	75	3,693.39	8	105.063	2	60,098.5	52
Environment (E)	2,792.16	14	512,187	8	39,649.51	83	2,364.438	49	31,536.8	27
G × E	1,841.56	10	613,589	10	4,510.99	9	2,382.437	49	15,986.6	14
Error	11,202.8	58	462,429	7	0	0	0	0	7,606	7
Total	19,341.16	100	6,248,407	100	47,853.89	100	4,851.937	100	115,227.9	100

*SS, sum of squares; Var.%, variation (%) of total variations.*

#### Genotypic Selection by Drought Tolerance Indices

The calculation of drought tolerance indices listed the top 10 genotypes exhibiting the highest distinct values for MP, GMP, and STI as CH39/08 (G20), CH40/09 (G19), K-01211 (G12), DG-2017 (G17), K002-10 (G9), 09AG006 (G26), K-01241 (G1), D-13012 (G33), CH15/11 (G27), and K- K-01308 (G2). These are categorized as Group B genotypes, indicating that these are the best performers in a non-stressed environment. A ranking of genotypes according to all drought tolerance indices is presented in Supplementary Table 4.

On the other hand, the top 10 genotypes with low TOL and SSI were BRC-457 (G35), CM584/09 (G38), DG-2017 (G17), D-13012 (G33), K-01211 (G12), CH39/08 (G20), CH10/08 (G19), CH56/09 (G13), Noor2013 (G11), and CH55/09 (G10). These genotypes are suggested to be the best genotypes specifically adapted to be cultivated in drought-stressed environments with better yield performance, i.e., Group C. Genotypes common to groups B and C are CH39/08 (G20), CH10/08 (G19), K-01211 (G12), and DG-2017 (G17), and thus fit into Group A.

TG12K10 (G6), CM877/10 (G5), TG12K02 (G7), CH15/11 (G27), Pb2008 (G22), K-01248 (G4), D-13011 (G36), DCD (G21), CH61/09 (G14), TG12K-07 (G16), and Noor 2013 (G11) were showing highest TOL and SSI values indicated as the most drought-susceptible genotypes with poor yield performance in drought stress conditions. Among these, TG12K10 (G6), TG12K02 (G7), CH61/09 (G14), CM877/10 (G5), DCD (G21), and D-13011 (G36) showed 68, 47, 35, 49, 34, and 35% yield reduction, respectively, due to drought and ranked 40, 39, 35, 25, 27, and 21 in GMP and STI, and may fall in Group D with other genotypes ranked least in MP, GMP, and STI. Moreover, CH15/11 (G27), K-01248 (G4), and TG12K-07 (G16) showed 35, 35, and 30% yield reduction, in that order, but ranked as 10, 13, and 15 in the GMP ranking list. Listing is based on the yield performance; genotypes are listed as low performing in comparison to other lines in the tested genotypes.

#### Correlation Among Phenotypes

**FIGURE 9:**
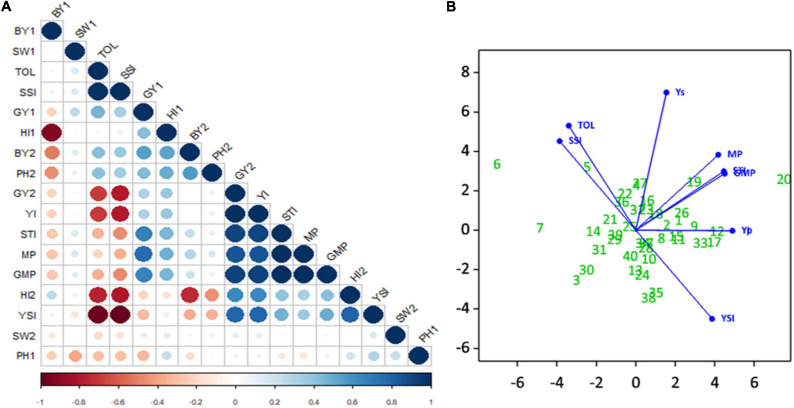
Experiment 2; **(A)** Correlation Matrix of traits and drought stress indices i.e., *biological yield* (BY), *seed weight* (SW), Stress tolerance Index (TOL), Stress Susceptibility Index (SSI), *seed yield* (SY), *harvest index* (HI), Yield Index (YI), Stress Tolerance Index (STI), Mean Productivity Index (MP), and Yield Stability Index under non-stressed (1) and stressed (2) conditions. Positive **correlations** are displayed in blue and **negative correlations** in red color. Color intensity and the size of the circle are proportional to the **correlation coefficients.** In the right side of the **correlogram,** the legend color shows the **correlation coefficients** and the corresponding colors. **(B)** Principle Component Biplot of drought tolerance indices and seed yield under simulating stressed and non-stressed conditions.

Evaluation of correlation coefficients showed a positive and significant correlation (*r*^2^ = 0.30) between seed yield under non-stress (Yp) and stress (Ys) environments, but the correlation coefficient was very low. A positive and significant correlation of Yp was observed with BY2, HI1, SW1, PH2, MP, GMP, STI, TOL, SSI, and YI. Also noted, Ys had a significant positive correlation with HI2, MP, GMP, STI, YI, and YSI. The correlations of TOL and SSI with Ys were negative and highly significant. Indices MP, GMP, STI, and YI had positive and highly significant correlation with each other; similarly, TOL was positively correlated with SSI. The highest positive correlation was observed between Ys and YI, and the highest negative correlation was observed between SSI and YSI. Seed weight did not show any significant correlations with other phenotypic traits (Figure 9A).

#### Principal Component Biplot Analysis

The principal component analysis simplifies the complex data by transforming the number of correlated variables into a smaller number of variables called principal components.

The principal component biplot (Figure 9B) shows that STI, MP, and GMP have a strong positive correlation with Yp and Ys, which indicates that these indices can be used to select high yielding genotypes in both conditions. However YI, YSI had strong positive correlation with Ys only, and this can identify superior genotypes for stressed environments. A strong positive correlation was observed between SSI and TOL. Moreover, SSI and TOL indices revealed a positive correlation with Yp and had a low negative correlation with STI, MP, GMP, and a strong negative correlation with Ys, YI, and YSI. Genotypes far from the origin were those more selectively influenced by these indices, and genotypes near the origin were less likely to be influenced by these indices; for instance, Yp, Ys, and STI, MP, GMP, YI indices selected with more confidence the genotypes CH39/08 (G20), CH40/09 (G19), K-01211 (G12), DG-2017 (G17) as better performer. SSI and TOL identified TG12K10 (G6), G12K02 (7), and K-01216G5 (G8) as the most sensitive to drought.

### Ranking of Drought Tolerant Genotypes in Natural and Simulating Stressed and Non-stressed Environments

Top 20 genotypes ranked in Experiments 1 and 2 for natural and simulating stressed and non-stressed environments are presented in Table 6. This can provide a broader view of the results obtained in this study and genotypes with consistent performance will be highlighted and selected for future use. Mostly genotypes ranked in both experiments have almost common places like CH39/08, CH40/09, and K-01211 were among the top genotypes in both experiments on the basis of seed yield. Similarly, genotypes TG12K10, TG12K02, and CM877/10 were found poor in producing seed yield in both experiments. Few genotypes behaved differently like DG-2017 was a low-yielding genotype in Punjab, due to severe infection by blight disease caused by *Ascochyta rabiei*, but ranked high in yield performance in Experiment 2.

**TABLE 6:** Top 20 genotypes selected from 2 year’s field conditioned drought stress experiments on the basis of AMMI selections, genotype selection indices (GSIs), and drought tolerance indices.

**Experiment 1**									**Experiment 2**					
**Non-stress**			**Stress**			**GSI-Overall**			**Non-stress**			**Stress**		
**Rank**	**Code**	**Genotype**	**Rank**	**Code**	**Genotype**	**Rank**	**Code**	**Genotype**	**Rank**	**Code**	**Genotype**	**Rank**	**Code**	**Genotype**
1	3	DCD	1	36	*TG12K10*	1	48	CH28/07	1	**20**	**CH39/08**	1	**6**	***TG12K10***
2	11	BRC457	2	37	*K01248*	2	2	CH39/08	2	19	CH40/09	2	5	*CM877/10*
3	73	CH15/11	3	41	K1221	3	11	BRC457	3	12	K-01211	3	7	*TG12K02*
4	10	D14005	4	44	*CM616/10*	4	73	CH15/11	4	17	DG-2017	4	22	Pb2008
5	18	K01216	5	45	K01338	5	3	DCD	5	9	K002-10	5	27	CH15/11
6	77	CH74/10	6	19	K00210	6	10	D14005	6	26	09AG006	6	36	D-13011
7	9	D13036	7	52	CH2/11	7	18	K01216	7	33	D-13012	7	14	CH61/09
8	20	CH55/09	8	54	CH13/11	8	20	CH55/09	8	1	*K-01241*	8	4	*K-01248*
9	22	CH56/09	9	62	PB2000	9	42	K01219	9	2	*K-01308*	9	21	DCD
10	17	D13030	10	64	CH23/00	10	77	CH74/10	10	27	CH15/11	10	16	TG12K-07
11	8	D13012	11	42	K01219	11	57	CH63/11	11	15	CH74/08	11	32	**Bittel-2016**
12	12	D13011	12	53	CH3/11	12	41	*CM616/10*	12	11	**Noor2013**	12	31	D-13036
13	78	BK2011	13	60	CH54/07	13	63	D07509	13	4	*K-01248*	13	39	D-13030
14	14	D13029	14	63	D07509	14	19	K00210	14	16	TG12K-07	14	23	CH49/09
15	29	CH77/08	15	68	CH2016	15	1	CH40/09	15	18	QG-1	15	29	AZC
16	2	CH39/08	16	51	CH1/11	16	49	CH10/08	16	23	CH49/09	16	25	CH28/07
17	76	BKK 2174	17	56	CH28/10	17	74	K850	17	8	K-01216	17	30	NIFA-1
19	1	CH40/09	18	57	CH63/11	18	12	D13011	18	32	**Bittel-2016**	18	3	*K-01242*
22	49	CH10/08	19	13	CH32/10	19	60	CH54/07	19	22	Pb2008	19	18	QG-1
24	56	CH28/10	20	48	CH28/07	20	21	K01211	20	36	D-13011	20	19	CH40/09
29	48	CH28/07	21	58	CH61/10	21	17	D13030	21	37	CH32/10	21	34	D-14005

*Low yielding and **cultivar check** genotypes are in *italics* and **bold**, respectively. GSI, genotypic selection index.*

## Discussion

### Drought Tolerance Evaluation of Advanced Breeding Lines Under Varying Natural Environments Should Be a Requirement Before Variety Approval

Drought-tolerance evaluation has been one of most studied traits in chickpea, as about 90% of chickpea is grown rainfed and dependent on left-over moisture from rainfall in the soil (Kumar and Abbo, 2001). Over a hundred studies done using drought-imposed yield reduction as a drought-tolerance measure showed significant variations in their results (Daryanto et al., 2015). Therefore, the selection of genotypes to be grown as cultivars needs to be evaluated for compliance with not only their yield under drought-stressed and non-stressed environments but also adaptation to their growing environments. Drought evaluation methods based on small-scale laboratory experiments in pots or tunnels are suitable for studying many drought-tolerance-related traits such as root architecture, vegetative growth rate, etc., but it is often seen that it is difficult, if not impossible, to reproduce laboratory results under field conditions. However, it is crucial to know how environments affect genotypes and, on this basis, to identify environment(s) unsurpassed for genotypes. Many studies have been carried out worldwide to evaluate genotype–environment interactions, e.g., Dehghani et al. (2010), Funga et al. (2017), Mohammadi et al. (2017), and Kaloki et al. (2019). In Pakistan. several research articles have been published on the subject, e.g., Arshad et al. (2003), Bakhsh et al. (2006), Atta et al. (2009), and Shah et al. (2020).

### Evaluation of Drought Tolerance in Naturally Stressed and Non-stressed Environments

In this study, selected environments were situated in the chickpea growing areas, and most of the chickpea cultivation in Punjab is concentrated on the marginal lands in Thal and Cholistan desert regions. Crop season for chickpea is winter in this region, flowering initiate in February, and most of flowering completes in March and pod setting simultaneously. Weather and moisture conditions in these months are critical for chickpea plant development and determines final yield. Eighty diverse chickpea genotypes were characterized for their drought-stress tolerance in varying natural growth environments, (i) with multi-environment trials in five rainfed areas as the drought-stressed conditions, and irrigated environment as non-stressed conditions, (ii) the 40 best-performing genotypes from multi-environment trials along with checks were re-evaluated for their drought-tolerance in natural field conditions by a spring-sowing experiment as the stressed simulating environment and winter sowing, which is normal growing season in Pakistan as non-stressed environment.

Trellis plots and analysis of variance by both AMMI and GLM models equally established that phenotypic traits GY, NPB, NSB, NOP, and NOS showed significant variations among environments; however, 100 SW and PH were stable, which means that environmental effects were the lowest on these traits. The non-stressed environment fared the best among all, which revealed that rainfed environments have enforced ample drought stress on plants and, hence, hampered their growth. Moreover, drought score measured by visual scoring was also minimum in non-stressed environment (Figure 3), thus implying that the difference in phenotypic observations recorded in rainfed environments was due to plants experiencing drought stress; Hintsa and Abay (2013) and Erdemci (2018) also reported similar findings.

A strong high positive correlation in NOP, NOS, and SY was observed. Moreover, SY and NOS were reduced when drought score increased (Figure 4A). Seed yield (SY) produced by individual genotypes was higher in the non-stressed environment (E5) compared to the stressed environments (Figure 5). Some genotypes performed well in most of the environments; however, some produced the highest mean seed yield at the non-stressed environment (E5). Similarly, some genotypes produced better mean seed yield in stressed environments (Figure 5). Previous studies also show that genotype behavior varied across environments (Kendal et al., 2016).

GEI plays an important role in the variable performance of the same genotype in different environments. The presence of strong GEI leads to cross over interactions or reversal of genotype ranks for trait variable such as yield in different environment (Yan and Tinker, 2006). The main effects are changes in the relative response and interaction of the genotypes in different environments. The main effects for genotypes and environment were extracted with AMMI model, which combines standard analysis of variance (ANOVA) with principal component analysis. As per good practice, GLM was applied to our data in addition to AMMI to compare analytical competitiveness of GLM with special software-based AMMI analysis. Also we have used GLM to identify main effects as an alternative to AMMI model. It measures variations contributed by genotypes, environment, and G × E interactions for each response variable by combined ANOVA and linear regression. GGE biplots are useful visualization tools that help identify which genotypes perform best for specific environments, and also which genotypes are more broadly adapted.

Upon GEI analysis, genotypes produced similar yields across environments clustered together. However, environments E4, E6, and E1 shared similar attributes. Environments E4 and E6 elicited weak, whereas E5 (non-stressed environment) elicited strong interactive forces on genotypes. Genotypes CH28/07 (G48) and D-07509 (G63) were influenced by GEI at most. G48 was observed specifically adapted to E3; similarly, D-14005 (G10), BRC-457 (G11), CH15/11 (G73), G18 (K-01216), and CH55/09 (G20) were specifically adapted and positively correlated to the non-stressed environment, i.e., E5. The first four AMMI genotype selections in each environment extracted from the AMMI main effects biplot (Figure 6A) are presented in Table 3.

The which-one-where biplot of seed yield contained three mega environments (ME), where ME3 consists of only non-stressed environment (Figure 6C). The comparison biplot showed that E4 shared more attributes with E5 than with others. Genotypes DCD (G3), CH15/11 (G73), D-14005 (G10), BRC-457 (G11), K-01216 (G18), and CH55/09 (G20) are likely to be ideal genotypes in the ideal environment E5 in terms of achieving higher mean seed yield and good stability showing by low IPCA score (Figure 6D). Ranking biplot ranked genotypes in each environment by their yield performance.

There are other manual methods used to establish the stability and yield potential of genotypes in multi-location replicated yield trials. The AMMI stability value (ASV) is a measure to estimate genotype stability; a lower ASV shows how more stable is the genotype (Purchase et al., 2000). However, the stability value may not be the only criteria for selection because stable genotypes may not be performing well in the area of producing seed yield. It was then proposed by Kang (1993) to use a selection criterion that takes into account both yield and stability. The genomic selection index (GSI), proposed by Farshadfar et al. (2011) renamed as “genotype selection index (GSI)” is based on AMMI stability value derived from IPCA sum of squares and scores and can be manually calculated to evaluate genotypes for both yield and stability. A lower value of GSI indicates desirable genotypes with high mean yield and stability. We identified that the GSI ranking is comparable to the AMMI model-based ranking conducted by GenStat software, GenStat-VSN International (2020) (Supplementary Tables 2, 3). Both identified the same top-most stable and high-yielding genotypes in all environments and ranked the same genotypes as low-yielding in comparison to the mean yield and as relatively less stable.

The AMMI model-based analyses were conducted extensively around the globe. A few references from the past and present that exhibited similar findings are being reviewed here in order to support the work conducted in this study: Bhardwaj et al. (2017), Mohammadi et al. (2017), Maqbool et al. (2017), Mathobo and Marais (2017), Oral et al. (2017), Das et al. (2018), Erdemci (2018), and Oral (2018).

### Evaluation of Drought Tolerance in Simulating Stressed and Non-stressed Environments

In Experiment 2, drought stress was imposed due to rise in temperature, decline in humidity, and increased day lengths in spring-sown chickpea. Winter and spring sowings were termed as non-stressed and stressed environments, respectively (Anwar et al., 2003). Winter-sown chickpea experienced a longer vegetative and reproductive period compared to spring-sown chickpea and, hence, produced larger leaf area and BY, which consequently resulted in the reduction in harvest index. Relatively, there are fewer reports about the comparison of chickpea growth in winter and spring sowings than drought-tolerance studies in winter. Our results endorsed the previous findings of Singh et al. (1987, 1998) and Toker et al. (2007).

Analysis of variance revealed that seed yield, biological yield, and plant height were more influenced by genotypic effects than by environment, and GEI indicates the germplasm tested was genetically diverse (Table 5).

It is established through many studies, e.g., Fischer and Maurer (1978) and Fernandez (1992), as the pioneer, and more recently, El-Hashash and Agwa (2018) that drought tolerance indices are the best criteria for the selection of discriminating, high-yielding drought-tolerant genotypes.

Results of many studies have also demonstrated that STI, GMP, and MP indices are the most relevant for the selection of drought-tolerant genotypes for both non-stressed and stressed environments as reported by Sanjari and Yazdansepas (2008) and Karimizadeh and Mohammadi (2011). Moreover, Golabadi et al. (2006) demonstrated that low SSI and TOL can also be used for the selection of superior genotypes for drought-prone areas, which can withstand drought stress better than other genotypes with a reasonable seed yield production. In this study, evaluation of correlation coefficients and principal component analysis showed that indices STI, MP, and GMP, due to strong positive correlation with Yp and Ys, can be used to select high-yielding genotypes in both conditions. However, YI and YSI had strong positive correlation with Ys only, and this can identify superior genotypes for stressed environments. The results obtained by principal component analysis confirmed the results obtained from drought-tolerance indices and correlation-based ranking of genotypes. El-Hashash and Agwa (2018) reported similar results.

## Conclusion and Prospects

Drought evaluation in rainfed environments or in multi-location trials leads us to conclude that it is important to select superior genotypes with improved yield in a range of environments where these are going to be cultivated. We were able to categorize the genotypes under study into groups according to their performance. We identified genotypes performing differentially well in stressed and non-stressed environments, and those performed consistently well in both environments. Ranks in each environment and GSI will help breeders select genotypes of their choice for further use as variety or pre-breeding germplasm.

Drought stress evaluation in winter- and spring-sown chickpea genotypes proved that a high-grade drought stress was imposed on plants sown in spring, which leads to differentiate clearly the level of stress by looking into the change in growth parameters. Seed yield was set as the drought stress determinant, and we used various mathematical and statistical implications to extract the identification of genotypes performing well in non-stressed and stressed conditions. The results concluded that this strategy was very helpful in identifying high-yielding drought-tolerant genotypes because we found that a few top genotypes identified for each environment in multi-location trials were also on top in the spring-sown drought stress experiments. MP, GMP, and YI indices were more effective in identifying high-yielding cultivars in diverse water scarcity. It was observable that the chickpea germplasm in Pakistan has a reasonable level of genotypic diversity.

Regarding the technical evaluation for drought-tolerance in chickpea, we will recommend that spring-sown imposition of stress is effective in identifying drought-tolerant genotypes with high yield prior to testing them in multi-location trials, which were ultimately required for approving variety with broad and specific adaptability for growing environments. This will preserve the time and resources, which may be considered in a country of limited resources such as Pakistan. It can also be concluded that a breeder may apply a combination of statistical techniques for authentic genotype selection procedure. In the changing climatic scenario, this study provides useful information for agricultural planning, crop modeling, and research directions for development of drought-tolerant legume species to improve adaptation and resilience of agricultural systems in the drought-prone regions of the world.

## Data Availability Statement

The original contributions presented in the study are included in the article/Supplementary Material, further inquiries can be directed to the corresponding author/s.

## Author Contributions

AA carried out the planning, execution, and technical guidance of this study, and contributed to the data analysis, the interpretation of results, and the preparation and writing of this manuscript. NP, MW, and IW contributed to the execution of field experiments, data recording, and analysis. TS provided the material used in this study, managed the out-station trials, and dispensed technical guidance throughout this study. RA contributed to the data analysis and the writing of the manuscript. All the authors contributed to the article and approved the submitted version.

## Conflict of Interest

The authors declare that the research was conducted in the absence of any commercial or financial relationships that could be construed as a potential conflict of interest.
