# Supplementary material for: A Comparative Study for Assessing the Drought-Tolerance of Chickpea Under Varying Natural Growth Environments
Source: Front Plant Sci. 2021 Feb 15;11:607869. doi: 10.3389/fpls.2020.607869 (PMC7928316; doi:10.3389/fpls.2020.607869)
Supplement: Supplementary file 1 [file Table_1.pdf]

Supplementary Table: 1 Mean yield (kg/ha) of advance breeding lines in preliminary yield trials conducted in multi environments across Pakistan

Trials	Advance Lines	Mean Yield (kg/hectare)	Environments
I	CH19/07, 11030, D-10008, NIFA-3, Pb-2008, SL-05-64,CH49/09, D-09027, CH28/07, 09AG006, TG-1221, 12011, DCD, CH40/09, TG×228, TG×220, CH17/08, CH10/08, CH39/08,D-0039, CM 1026/09, Bittle-16, *NIFA-1*, NIFA-2, AZC, TG-1218	2587, 2586, 2617, 2187, 2717, 2236, 2717, 2493, 2676, 2636,<br>2543, 2475, 2728, 2817, 2435, 2362, 2616, 2512, 2752, 2480, 2612, 2710, 1581, 2066, 2048, 2540	Faisalabad,<br>Karak, Bhakkar,<br>Kallur Kot (Pb), Islamabad (Fed), Peshawar, D.I.Khan (KPK), Larkana (Sindh)
II	D-13011, D-13012, D-13029, D-13030, D-13031, D-13036, D-14005, D-14008,D-14013, D-14014, BRC-424, BRC-457, TG-1306, CH19/10, CH35/10, CH32/10, CM1036/09, *CM584/09*, Pb.2008, Bittal 2016	2131, 2241, 2073, 1764, 1894, 2248, 2181, 2048, 1952, 1928, 19722173, 2067, 1923, 1992, 2075, 1898, 1881, 2073, 2112	Faisalabad<br>Kallur Kot<br>Karor, Rakh-uttra,<br>Bhakkar, Bahawalpur (Pb)
III	*QG-4*, TG12K-07, CH55/09, *QG-3*, K-1209<br>CH77/08, CH61/09, *DG-2017*, K-01216 K-01211, *QG-1*, Noor 2013, CH74/08, *TG12K-01*, K002-10, BKK2174, CH56/09, CH72/08, CH76/08	1613, 2173, 2250, 1663, 2126, 2137, 2207, 1562, 2275, 2224 1549, 2038, 2189, 1912, 2259 2161, 2209, 2165, 2146	Faisalabad, Bhakkar, Chakwal, Kallur Kot (Pb) Islamabad (Fed), Karak DI Khan (KPK), Tando Jam, Larkana (Sindh)
IV	*K-01241, K-01308, K-01242, K-01248, K-1221, K-01302, K-01250, CM877/10, CM616/10, K-01219, K-01240, K-01338,* Noor-2013, *TG12K05, TG12K01, CM545/10, TG12K10, TG12K02*	1660, 1636, 1567, 1566, 1551, 1539, 1534, 1512, 1491, 1489 1461, 1438, 1390, 1351, 1320 1316, 1209, 1048	Faisalabad,<br>Kallur Kot, Rakhuttra,<br>Bhakkar,<br>Bahawalpur, Chakwal,<br>Fateh Jang

Sequence of genotypes and means are in order from Left to Right. Commercial varieties are shown in bold. Low Yielding are shown in Italics.
