# Supplementary material for: A Comparative Study for Assessing the Drought-Tolerance of Chickpea Under Varying Natural Growth Environments
Source: Front Plant Sci. 2021 Feb 15;11:607869. doi: 10.3389/fpls.2020.607869 (PMC7928316; doi:10.3389/fpls.2020.607869)
Supplement: Supplementary file 2 [file Table_2.pdf]

Supplementary Table: 2 Rankings of 80 chickpea genotypes on AMMI-Estimates for mean seed yield (g/plot) in six environments

Code	Genotype	Desc.	Status	Source	Ranked on AMMI-Estimates (g/plot)					
					Env-1	Env-2	Env-3	Env-4	Env-5	Env-6
**G1**	CH40/09	Desi	Adv. line	NIAB-Fsd	G64	G73	G48	G48	G3	G49
**G2**	CH39/08	Desi	Adv. line	NIAB-Fsd	G42	G10	G2	G2	G11	G14
**G3**	DCD	Desi	Adv. line	AZRI-DIK	G53	G14	G41	G49	G73	G48
**G4**	CH49/09	Desi	Adv. line	NIAB-Fsd	G52	G11	G49	G11	G10	G73
**G5**	NIFA-2	Desi	Adv. line	NIFA-PSR	G62	G9	G63	G8	G18	G16
**G6**	AZC	Desi	Adv. line	AZRI-BWP	G14	G16	G19	G10	G77	G10
**G7**	*NIFA-1*	Desi	Adv. line	NIFA-PSR	G16	G12	G57	G9	G9	G42
**G8**	D-13036	Desi	Adv. line	AARI-Fsd	G23	G20	G74	G73	G20	G9
**G9**	D-13012	Desi	Adv. line	AARI-Fsd	G73	G8	G60	G3	G22	G8
**G10**	D-14005	Desi	Adv. line	AARI-Fsd	G71	G18	G1	G1	G17	G57
**G11**	BRC-457	Desi	Adv. line	------------	G21	G49	G45	G19	G8	G53
**G12**	D-13011	Desi	Adv. line	AARI-Fsd	G49	G17	G44	G14	G12	G23
**G13**	CH32/10	Desi	Adv. line	NIAB-Fsd	G60	G21	G51	G17	G78	G62
**G14**	D-13029	Desi	Adv. line	AARI-Fsd	G68	G77	G36	G76	G14	G2
**G15**	D-13031	Desi	Adv. line	AARI-Fsd	G57	G3	G58	G12	G29	G12
**G16**	*CM584/09*	Desi	Adv. line	NIAB-Fsd	G63	G23	G54	G13	G2	G58
**G17**	D-13030	Desi	Adv. line	AARI-Fsd	G10	G29	G37	G4	G76	G51
**G18**	K-01216	Kabuli	Adv. line	AARI-Fsd	G12	G15	G8	G16	G4	G17
**G19**	K002-10	Kabuli	Adv. line	AARI-Fsd	G58	G78	G13	G56	G1	G21
**G20**	CH55/09	Kabuli	Adv. line	NIAB-Fsd	G72	G4	G76	G57	G33	G52
**G21**	K-01211	Kabuli	Adv. line	AARI-Fsd	G9	G48	G59	G78	G16	G11
**G22**	CH56/09	Kabuli	Adv. line	NIAB-Fsd	G51	G53	G24	G18	G49	G63
**G23**	CH61/09	Kabuli	Adv. line	NIAB-Fsd	G69	G72	G56	G59	G13	G68
**G24**	CH74/08	Kabuli	Adv. line	NIAB-Fsd	G20	G2	G39	G54	G56	G64
**G25**	TG12K-07	Kabuli	Adv. line	AZRI-Bhk	G8	G42	G25	G77	G21	G60
**G26**	CH72/08	Kabuli	Adv. line	NIAB-Fsd	G70	G13	G68	G58	G80	G20
**G27**	BKK2174	Kabuli	Adv. line	NIAB-Fsd	G28	G56	G14	G51	G15	G74
**G28**	CH76/08	Kabuli	Adv. line	NIAB-Fsd	G74	G58	G4	G25	G27	G4
**G29**	CH77/08	Kabuli	Adv. line	NIAB-Fsd	G15	G76	G9	G80	G48	G13
**G30**	K-01209	Kabuli	Adv. line	AARI-Fsd	G26	G71	G42	G20	G61	G15
**G31**	DG-2017	Kabuli	Adv. line	Dokri-Lrk	G48	G25	G16	G27	G47	G41
**G32**	*QG-1*	Kabuli	Adv. line	QAARI-Lrk	G50	G62	G11	G41	G59	G25
**G33**	*K-01241*	Kabuli	Adv. line	AARI-Fsd	G17	G22	G80	G22	G19	G56
**G34**	*K-01308*	Kabuli	Adv. line	AARI-Fsd	G67	G61	G62	G15	G72	G54
**G35**	*K-01242*	Kabuli	Adv. line	AARI-Fsd	G25	G57	G17	G74	G25	G18
**G36**	*K-01248*	Kabuli	Adv. line	AARI-Fsd	G4	G59	G40	G47	G54	G59
**G37**	K-1221	Kabuli	Adv. line	AARI-Fsd	G61	G52	G64	G29	G6	G28
**G38**	*K-01302*	Kabuli	Adv. line	AARI-Fsd	G41	G51	G38	G36	G5	G76
**G39**	*K-01250*	Kabuli	Adv. line	AARI-Fsd	G7	G80	G27	G33	G66	G29
**G40**	*CM877/10*	Kabuli	Adv. line	NIAB-Fsd	G24	G26	G47	G37	G55	G77
**G41**	*CM616/10*	Kabuli	Adv. line	NIAB-Fsd	G5	G27	G28	G23	G26	G19
**G42**	*K-01219*	Kabuli	Adv. line	AARI-Fsd	G29	G54	G10	G66	G58	G78
**G43**	*K-01240*	Kabuli	Adv. line	AARI-Fsd	G35	G6	G23	G24	G23	G24

**G44**	*K-01338*	Kabuli	Adv. line	AARI-Fsd	G6	G5	G79	G21	G79	G1
**G45**	*TG12K10*	Kabuli	Adv. line	AZRI-Bhk	G66	G66	G12	G55	G34	G37
**G46**	*TG12K02*	Kabuli	Adv. line	AZRI-Bhk	G18	G1	G70	G6	G43	G72
**G47**	09 AG 006	Desi	Adv. line	NIAB-Fsd	G45	G28	G66	G79	G50	G71
**G48**	CH28/07	Desi	Adv. line	NIAB-Fsd	G13	G68	G78	G68	G75	G66
**G49**	CH10/08	Desi	Adv. line	NIAB-Fsd	G56	G50	G55	G42	G51	G6
**G50**	CM1036-09	Desi	Adv. line	NIAB-Fsd	G54	G33	G30	G28	G71	G80
**G51**	CH 1/11	Desi	Adv. line	NIAB-Fsd	G59	G55	G35	G63	G28	G50
**G52**	CH 2/11	Desi	Adv. line	NIAB-Fsd	G37	G67	G73	G62	G67	G27
**G53**	CH 3/11	Desi	Adv. line	NIAB-Fsd	G77	G19	G15	G44	G57	G5
**G54**	CH13/11	Desi	Adv. line	NIAB-Fsd	G65	G47	G6	G5	G36	G26
**G55**	CH50/11	Desi	Adv. line	NIAB-Fsd	G11	G79	G33	G34	G37	G45
**G56**	CH28/10	Desi	Adv. line	NIAB-Fsd	G38	G24	G53	G53	G53	G3
**G57**	CH63/11	Kabuli	Adv. line	NIAB-Fsd	G30	G74	G3	G50	G24	G55
**G58**	CH61/10	Kabuli	Adv. line	NIAB-Fsd	G55	G37	G34	G39	G68	G70
**G59**	CH69/09	Kabuli	Adv. line	NIAB-Fsd	G2	G34	G52	G45	G46	G36
**G60**	CH54/07	Desi	Adv. line	NIAB-Fsd	G78	G75	G50	G26	G62	G61
**G61**	Karak-2	Desi	Adv. line	ARS-Krk	G76	G7	G5	G60	G42	G35
**G62**	PB-2000	Desi	Adv. line	AARI-Fsd	G27	G35	G29	G61	G7	G79
**G63**	D-07509	Desi	Adv. line	AARI-Fsd	G46	G36	G69	G38	G30	G67
**G64**	CH-23/00	Kabuli	Adv. line	NIAB-Fsd	G80	G43	G18	G40	G35	G38
**G65**	ILC-3279	Desi	Trad. line	ICRISAT	G79	G30	G32	G72	G40	G69
**G66**	Bittel-16	Desi	Cultivar	AARI-Fsd	G75	G46	G77	G30	G52	G47
**G67**	CM-88	Desi	Adv. line	NIAB-Fsd	G32	G41	G20	G35	G74	G44
**G68**	CH-2016	Desi	Adv. line	NIAB-Fsd	G36	G38	G22	G52	G38	G30
**G69**	Paidar-91	Desi	Cultivar	AARI-Fsd	G44	G60	G21	G43	G39	G7
**G70**	E-26	Desi	Trad. line	ICRISAT	G34	G70	G46	G67	G65	G33
**G71**	Karak-98	Desi	Adv. line	NIFA-PSR	G19	G63	G7	G46	G44	G22
**G72**	6153	Desi	Trad. line	ICRISAT	G1	G64	G26	G75	G41	G34
**G73**	CH15/11	Desi	Adv. line	NIAB-Fsd	G39	G65	G43	G7	G31	G39
**G74**	K-850	Desi	Trad. line	ICRISAT	G47	G69	G31	G70	G70	G46
**G75**	Aug 424	Desi	Cultivar	ICRISAT	G43	G39	G67	G71	G69	G75
**G76**	BKK 2174	Desi	Adv. line	NIAB-Fsd	G33	G44	G61	G69	G45	G43
**G77**	CH74/10	Kabuli	Adv. line	NIAB-Fsd	G22	G45	G75	G64	G32	G40
**G78**	BK-2011	Desi	Cultivar	AZRI-Bhk	G3	G40	G72	G31	G63	G65
**G79**	Noor-13	Kabuli	Cultivar	AARI-Fsd	G31	G32	G71	G32	G60	G32
**G80**	CM2008	Kabuli	Cultivar	NIAB-Fsd	G40	G31	G65	G65	G64	G31

Abbreviation: Descriptor (Desc.)
