# Supplementary material for: A Comparative Study for Assessing the Drought-Tolerance of Chickpea Under Varying Natural Growth Environments
Source: Front Plant Sci. 2021 Feb 15;11:607869. doi: 10.3389/fpls.2020.607869 (PMC7928316; doi:10.3389/fpls.2020.607869)
Supplement: Supplementary file 3 [file Table_3.pdf]

Supplementary Table 3: Seed mean yield (SYm), Rank of yield (RY), AMMI stability value (ASV), ranks of ASV (RASV) and Genotype Selection Index (GSI) based ranking of 80 chickpea genotypes

S #	Code	Genotype	GYm	RY	ASV	RASV	GSI	S #	Code	Genotype	GYm	RY	ASV	RASV	GSI
**1**	**G48**	CH28/07	658	1	13	7	8	**41**	G68	CH-2016	482	37	6	40	77
**2**	**G2**	CH39/08	605	6	10	14	20	**42**	G61	Karak-2	412	60	9	22	82
**3**	**G11**	BRC-457	591	9	11	12	21	**43**	G4	CH49/09	527	16	3	67	83
**4**	**G73**	CH15/11	627	3	9	19	22	**44**	G36	*K-01248*	437	50	7	35	85
**5**	**G3**	DCD	513	25	16	4	29	**45**	G13	CH32/10	521	20	3	68	88
**6**	**G10**	D-14005	614	5	9	24	29	**46**	G33	*K-01241*	424	57	7	33	90
**7**	**G18**	K-01216	520	21	11	11	32	**47**	G15	D-13031	499	34	4	57	91
**8**	**G20**	CH55/09	526	17	10	16	33	**48**	G37	K-1221	454	45	5	48	93
**9**	**G42**	*K-01219*	523	18	9	20	38	**49**	G54	CH13/11	499	33	4	60	93
**10**	**G77**	CH74/10	501	32	12	10	42	**50**	G56	CH28/10	513	24	3	69	93
**11**	**G57**	CH63/11	552	13	8	30	43	**51**	G80	**CM2008**	475	40	4	53	93
**12**	**G41**	*CM616/10*	479	39	14	5	44	**52**	G27	BKK2174	467	41	4	55	96
**13**	**G63**	D-07509	461	42	17	2	44	**53**	G70	E-26	372	70	8	26	96
**14**	**G19**	K002-10	509	27	9	18	45	**54**	G47	09 AG 006	430	56	6	42	98
**15**	**G1**	CH40/09	515	22	9	25	47	**55**	G69	Paidar-91	330	76	9	23	99
**16**	**G49**	CH10/08	650	2	5	47	49	**56**	G59	CH69/09	502	30	3	70	100
**17**	**G74**	K-850	488	36	10	15	51	**57**	G24	CH74/08	452	47	4	56	103
**18**	**G12**	D-13011	564	11	6	41	52	**58**	G26	CH72/08	422	58	6	46	104
**19**	**G60**	CH54/07	446	49	16	3	52	**59**	G67	CM-88	379	68	6	37	105
**20**	**G21**	K-01211	509	26	8	27	53	**60**	G25	TG12K-07	503	28	2	78	106
**21**	**G17**	D-13030	560	12	6	43	55	**61**	G39	*K-01250*	359	71	6	36	107
**22**	**G53**	CH 3/11	502	29	8	28	57	**62**	G65	ILC-3279	258	78	8	31	109
**23**	**G9**	D-13012	598	8	5	51	59	**63**	G75	Aug 424	335	75	6	39	114
**24**	**G51**	CH 1/11	527	15	6	45	60	**64**	G40	*CM877/10*	326	77	6	38	115
**25**	**G62**	PB-2000	502	31	8	29	60	**65**	G5	NIFA-2	435	51	3	65	116
**26**	**G14**	D-13029	625	4	4	58	62	**66**	G28	CH76/08	461	44	2	73	117
**27**	**G22**	CH56/09	432	54	12	8	62	**67**	G38	*K-01302*	383	67	4	54	121
**28**	**G52**	CH 2/11	461	43	9	21	64	**68**	G6	AZC	448	48	2	75	123
**29**	**G64**	CH-23/00	391	63	20	1	64	**69**	G32	*QG-1*	251	79	6	44	123
**30**	**G78**	**BK-2011**	499	35	7	32	67	**70**	G43	*K-01240*	337	74	5	49	123
**31**	**G45**	*TG12K10*	392	62	13	6	68	**71**	G66	**Bittel-16**	453	46	2	79	125
**32**	**G8**	D-13036	599	7	4	62	69	**72**	G50	CM1036/09	430	55	2	72	127
**33**	**G23**	CH61/09	522	19	5	50	69	**73**	G34	*K-01308*	389	65	4	63	128
**34**	**G72**	6153	434	52	10	17	69	**74**	G55	CH50/11	434	53	2	76	129
**35**	**G71**	Karak-98	400	61	12	9	70	**75**	G35	*K-01242*	386	66	3	66	132
**36**	**G16**	*CM584/09*	588	10	4	61	71	**76**	G79	**Noor-13**	419	59	2	74	133
**37**	**G29**	CH77/08	481	38	7	34	72	**77**	G7	*NIFA-1*	359	72	4	64	136
**38**	**G58**	CH61/10	534	14	4	59	73	**78**	G30	K-01209	377	69	3	71	140
**39**	**G76**	BKK 2174	514	23	5	52	75	**79**	G46	*TG12K02*	340	73	2	77	150
**40**	**G44**	*K-01338*	389	64	10	13	77	**80**	G31	DG-2017	227	80	1	80	160
