# Supplementary material for: A Comparative Study for Assessing the Drought-Tolerance of Chickpea Under Varying Natural Growth Environments
Source: Front Plant Sci. 2021 Feb 15;11:607869. doi: 10.3389/fpls.2020.607869 (PMC7928316; doi:10.3389/fpls.2020.607869)
Supplement: Supplementary file 4 [file Table_4.pdf]

Supplementary Table: 4 Ranking of Genotypes on Mean Productivity (MP), Geometric Mean Productivity (GMP), Drought tolerance Index (TOL), Stress Tolerance Index (STI), Stress Susceptibility Index (SSI), Yield Index (YI) and Yield Stability Index (YSI). *R denotes Rank.*

Code	Geno	GYp	GYs	YR (%)	RGYp	Code	Geno	Gyp	RGYs	Code	Geno	GYs	RMP	Code	Geno	MP	RGMP	Code	Geno	GMP
**1**	*K-01241*	1096	897	18	**1**	20	CH39/08	1296	**1**	20	CH39/08	1192	**1**	20	CH39/08	1244	**1**	20	CH39/08	1243
**2**	*K-01308*	1072	863	19	**2**	19	CH40/09	1232	**2**	12	K-01211	1000	**2**	19	CH40/09	1080	**2**	19	CH40/09	1069
**3**	*K-01242*	808	600	26	**3**	5	*CM877/10*	1216	**3**	17	DG-2017	992	**3**	12	K-01211	1043	**3**	12	K-01211	1042
**4**	*K-01248*	1184	770	35	**4**	27	CH15/11	1192	**4**	33	D-13012	952	**4**	17	DG-2017	1021	**4**	17	DG-2017	1021
**5**	*CM877/10*	1216	624	49	**5**	4	*K-01248*	1184	**5**	9	K002-10	944	**5**	9	K002-10	1016	**5**	9	K002-10	1013
**6**	*TG12K10*	1168	371	68	**6**	6	*TG12K10*	1168	**6**	19	CH40/09	928	**6**	26	09AG006	1008	**6**	26	09AG006	1002
**7**	*TG12K02*	952	500	47	**7**	22	Pb2008	1144	**7**	1	*K-01241*	898	**7**	1	*K-01241*	997	**7**	33	D-13012	994
**8**	K-01216	1024	848	17	**8**	16	TG12K-07	1136	**8**	26	09AG006	896	**8**	33	D-13012	995	**8**	1	*K-01241*	992
**9**	K002-10	1088	944	13	**9**	26	09AG006	1120	**9**	11	**Noor2013**	888	**9**	27	CH15/11	980	**9**	2	*K-01308*	962
**10**	CH55/09	945	800	15	**10**	36	D-13011	1112	**10**	15	CH74/08	881	**10**	4	*K-01248*	977	**10**	27	CH15/11	958
**11**	**Noor2013**	1031	888	14	**11**	23	CH49/09	1104	**11**	2	*K-01308*	863	**11**	2	*K-01308*	967	**11**	15	CH74/08	957
**12**	K-01211	1086	1000	8	**12**	1	*K-01241*	1096	**12**	8	K-01216	848	**12**	16	TG12K-07	964	**12**	11	**Noor2013**	957
**13**	CH56/09	896	760	15	**13**	18	*QG-1*	1096	**13**	18	*QG-1*	819	**13**	28	13029	964	**13**	4	*K-01248*	955
**14**	CH61/09	984	640	35	**14**	32	**Bittel-2016**	1096	**14**	35	BRC-457	817	**14**	15	CH74/08	960	**14**	28	13029	949
**15**	CH74/08	1040	880	15	**15**	9	K002-10	1088	**15**	10	CH55/09	800	**15**	11	**Noor2013**	959	**15**	16	TG12K-07	949
**16**	TG12K-07	1136	792	30	**16**	12	K-01211	1086	**16**	37	CH32/10	794	**16**	18	*QG-1*	958	**16**	18	*QG-1*	948
**17**	DG-2017	1050	992	6	**17**	2	*K-01308*	1072	**17**	16	TG12K-07	792	**17**	23	CH49/09	948	**17**	23	CH49/09	935
**18**	*QG-1*	1096	819	25	**18**	17	DG-2017	1050	**18**	23	CH49/09	792	**18**	8	K-01216	936	**18**	8	K-01216	932
**19**	CH40/09	1232	928	25	**19**	15	CH74/08	1040	**19**	28	13029	792	**19**	22	Pb2008	936	**19**	32	**Bittel-2016**	916
**20**	CH39/08	1296	1192	8	**20**	21	DCD	1040	**20**	38	*CM584/09*	792	**20**	32	**Bittel-2016**	931	**20**	22	Pb2008	913
**21**	DCD	1040	688	34	**21**	33	D-13012	1038	**21**	34	D-14005	784	**21**	5	*CM877/10*	920	**21**	36	D-13011	895
**22**	Pb2008	1144	728	36	**22**	25	CH28/07	1032	**22**	24	CH10/08	780	**22**	36	D-13011	916	**22**	37	CH32/10	888
**23**	CH49/09	1104	792	28	**23**	11	**Noor2013**	1031	**23**	4	*K-01248*	770	**23**	37	CH32/10	893	**23**	34	D-14005	880
**24**	CH10/08	888	780	12	**24**	8	K-01216	1024	**24**	27	CH15/11	770	**24**	25	CH28/07	889	**24**	25	CH28/07	877
**25**	CH28/07	1032	745	28	**25**	37	CH32/10	992	**25**	32	**Bittel-2016**	766	**25**	34	D-14005	886	**25**	5	*CM877/10*	871
**26**	09AG006	1120	896	20	**26**	39	D-13030	992	**26**	13	CH56/09	760	**26**	10	CH55/09	872	**26**	10	CH55/09	869
**27**	CH15/11	1192	769	35	**27**	34	D-14005	988	**27**	40	**BK-2011**	747	**27**	21	DCD	864	**27**	21	DCD	846

**28**	13029	976	792	19	**28**	14	CH61/09	984	**28**	25	CH28/07	746	**28**	39	D-13030	848	**28**	40	**BK-2011**	836
**29**	AZC	976	701	28	**29**	28	13029	976	**29**	22	Pb2008	728	**29**	40	**BK-2011**	841	**29**	39	D-13030	836
**30**	*NIFA-1*	848	620	27	**30**	29	AZC	976	**30**	36	D-13011	720	**30**	29	AZC	839	**30**	35	BRC-457	832
**31**	D-13036	928	657	29	**31**	7	*TG12K02*	952	**31**	39	D-13030	704	**31**	24	CH10/08	834	**31**	24	CH10/08	832
**32**	**Bittel2016**	1096	765	30	**32**	10	CH55/09	944	**32**	29	AZC	702	**32**	35	BRC-457	832	**32**	29	AZC	828
**33**	D-13012	1037	952	8	**33**	40	**BK-2011**	936	**33**	21	DCD	688	**33**	13	CH56/09	828	**33**	13	CH56/09	825
**34**	D-14005	988	784	21	**34**	31	D-13036	928	**34**	31	D-13036	658	**34**	14	CH61/09	812	**34**	38	*CM584/09*	808
**35**	BRC-457	848	816	4	**35**	13	CH56/09	896	**35**	14	CH61/09	640	**35**	38	*CM584/09*	808	**35**	14	CH61/09	794
**36**	D-13011	1112	720	35	**36**	24	CH10/08	888	**36**	5	*CM877/10*	624	**36**	31	D-13036	793	**36**	31	D-13036	781
**37**	CH32/10	992	794	20	**37**	30	*NIFA-1*	848	**37**	30	*NIFA-1*	621	**37**	6	*TG12K10*	770	**37**	30	*NIFA-1*	726
**38**	*CM584/09*	824	792	4	**38**	35	BRC-457	848	**38**	3	*K-01242*	601	**38**	30	*NIFA-1*	734	**38**	3	*K-01242*	697
**39**	D-13030	992	704	29	**39**	38	*CM584/09*	824	**39**	7	*TG12K02*	501	**39**	7	*TG12K02*	726	**39**	7	*TG12K02*	690
**40**	BK-2011	936	747	20	**40**	3	*K-01242*	808	**40**	6	*TG12K10*	371	**40**	3	*K-01242*	704	**40**	6	*TG12K10*	658

RTOL	Code	Genotype	TOL	RSTI	Code	Genotype	STI	RSSI	Code	Genotype	SSI	RYI	Code	Genotype	YI	RYSI	Code	Genotype	YSI
**1**	6	***TG12K10***	**797**	**1**	20	CH39/08	1.44	**1**	6	***TG12K10***	**2.8**	**1**	20	CH39/08	1.52	**1**	35	BRC-457	0.96
**2**	5	*CM877/10*	592	**2**	19	CH40/09	1.06	**2**	5	*CM877/10*	2	**2**	12	K-01211	1.28	**2**	38	*CM584/09*	0.96
**3**	7	*TG12K02*	451	**3**	12	K-01211	1.01	**3**	7	*TG12K02*	1.9	**3**	17	DG-2017	1.27	**3**	17	DG-2017	0.94
**4**	27	CH15/11	422	**4**	17	DG-2017	0.97	**4**	22	Pb2008	1.5	**4**	33	D-13012	1.21	**4**	12	K-01211	0.92
**5**	22	Pb2008	416	**5**	9	K002-10	0.96	**5**	27	CH15/11	1.5	**5**	9	K002-10	1.2	**5**	20	CH39/08	0.92
**6**	4	*K-01248*	414	**6**	26	09AG006	0.93	**6**	36	D-13011	1.4	**6**	19	CH40/09	1.18	**6**	33	D-13012	0.92
**7**	36	D-13011	392	**7**	33	D-13012	0.92	**7**	14	CH61/09	1.4	**7**	1	*K-01241*	1.14	**7**	24	CH10/08	0.88
**8**	21	DCD	352	**8**	1	*K-01241*	0.92	**8**	4	*K-01248*	1.4	**8**	26	09AG006	1.14	**8**	9	K002-10	0.87
**9**	14	CH61/09	344	**9**	2	*K-01308*	0.86	**9**	21	DCD	1.4	**9**	11	**Noor2013**	1.13	**9**	11	**Noor2013**	0.86
**10**	16	TG12K-07	344	**10**	27	CH15/11	0.85	**10**	16	TG12K-07	1.2	**10**	15	CH74/08	1.12	**10**	13	CH56/09	0.85
**11**	32	**Bittel-2016**	330	**11**	15	CH74/08	0.85	**11**	32	**Bittel-2016**	1.2	**11**	2	*K-01308*	1.1	**11**	15	CH74/08	0.85
**12**	23	CH49/09	312	**12**	11	**Noor2013**	0.85	**12**	31	D-13036	1.2	**12**	8	K-01216	1.08	**12**	10	CH55/09	0.85
**13**	19	CH40/09	304	**13**	4	*K-01248*	0.85	**13**	39	D-13030	1.2	**13**	18	*QG-1*	1.04	**13**	8	K-01216	0.83
**14**	39	D-13030	288	**14**	16	TG12K-07	0.84	**14**	23	CH49/09	1.2	**14**	35	BRC-457	1.04	**14**	1	*K-01241*	0.82
**15**	25	CH28/07	286	**15**	18	*QG-1*	0.84	**15**	29	AZC	1.2	**15**	10	CH55/09	1.02	**15**	28	13029	0.81
**16**	18	*QG-1*	277	**16**	23	CH49/09	0.81	**16**	25	CH28/07	1.1	**16**	37	CH32/10	1.01	**16**	2	*K-01308*	0.81
**17**	29	AZC	274	**17**	8	K-01216	0.81	**17**	30	*NIFA-1*	1.1	**17**	16	TG12K-07	1.01	**17**	37	CH32/10	0.8
**18**	31	D-13036	270	**18**	32	**Bittel-2016**	0.78	**18**	3	*K-01242*	1.1	**18**	23	CH49/09	1.01	**18**	26	09AG006	0.8
**19**	30	*NIFA-1*	227	**19**	22	Pb2008	0.77	**19**	18	*QG-1*	1	**19**	28	13029	1.01	**19**	40	BK-2011	0.8
**20**	26	09AG006	224	**20**	36	D-13011	0.74	**20**	19	CH40/09	1	**20**	38	*CM584/09*	1.01	**20**	34	D-14005	0.79

**21**	2	*K-01308*	209	**21**	37	CH32/10	0.73	**21**	34	D-14005	0.8	**21**	34	D-14005	1	**21**	19	CH40/09	0.75
**22**	3	*K-01242*	207	**22**	34	D-14005	0.72	**22**	40	BK-2011	0.8	**22**	24	CH10/08	0.99	**22**	18	*QG-1*	0.75
**23**	34	D-14005	204	**23**	28	13029	0.72	**23**	26	09AG006	0.8	**23**	4	*K-01248*	0.98	**23**	3	*K-01242*	0.74
**24**	1	*K-01241*	198	**24**	25	CH28/07	0.72	**24**	37	CH32/10	0.8	**24**	27	CH15/11	0.98	**24**	30	*NIFA-1*	0.73
**25**	37	CH32/10	198	**25**	5	*CM877/10*	0.71	**25**	2	*K-01308*	0.8	**25**	32	**Bittel-2016**	0.98	**25**	25	CH28/07	0.72
**26**	40	**BK-2011**	189	**26**	10	CH55/09	0.7	**26**	28	13029	0.8	**26**	13	CH56/09	0.97	**26**	29	AZC	0.72
**27**	28	13029	184	**27**	21	DCD	0.67	**27**	1	*K-01241*	0.7	**27**	40	BK-2011	0.95	**27**	23	CH49/09	0.72
**28**	8	K-01216	176	**28**	40	**BK-2011**	0.65	**28**	8	K-01216	0.7	**28**	25	CH28/07	0.95	**28**	39	D-13030	0.71
**29**	15	CH74/08	159	**29**	39	D-13030	0.65	**29**	15	CH74/08	0.6	**29**	22	Pb2008	0.93	**29**	31	D-13036	0.71
**30**	9	K002-10	144	**30**	35	BRC-457	0.64	**30**	10	CH55/09	0.6	**30**	36	D-13011	0.92	**30**	32	**Bittel-2016**	0.7
**31**	10	CH55/09	144	**31**	24	CH10/08	0.64	**31**	13	CH56/09	0.6	**31**	39	D-13030	0.9	**31**	16	TG12K-07	0.7
**32**	11	**Noor2013**	143	**32**	29	AZC	0.64	**32**	11	**Noor2013**	0.6	**32**	29	AZC	0.89	**32**	21	DCD	0.66
**33**	13	CH56/09	136	**33**	13	CH56/09	0.63	**33**	9	K002-10	0.5	**33**	21	DCD	0.88	**33**	4	*K-01248*	0.65
**34**	24	CH10/08	108	**34**	38	*CM584/09*	0.61	**34**	24	CH10/08	0.5	**34**	31	D-13036	0.84	**34**	14	CH61/09	0.65
**35**	20	CH39/08	104	**35**	14	CH61/09	0.59	**35**	33	D-13012	0.3	**35**	14	CH61/09	0.82	**35**	36	D-13011	0.65
**36**	12	K-01211	86	**36**	31	D-13036	0.57	**36**	20	CH39/08	0.3	**36**	5	*CM877/10*	0.8	**36**	27	CH15/11	0.65
**37**	33	D-13012	86	**37**	30	*NIFA-1*	0.49	**37**	12	K-01211	0.3	**37**	30	*NIFA-1*	0.79	**37**	22	Pb2008	0.64
**38**	17	DG-2017	58	**38**	3	*K-01242*	0.45	**38**	17	DG-2017	0.2	**38**	3	*K-01242*	0.77	**38**	7	*TG12K02*	0.53
**39**	38	*CM584/09*	32	**39**	7	*TG12K02*	0.44	**39**	38	*CM584/09*	0.2	**39**	7	*TG12K02*	0.64	**39**	5	*CM877/10*	0.51
**40**	35	BRC-457	31	**40**	6	*TG12K10*	0.4	**40**	35	BRC-457	0.2	**40**	6	*TG12K10*	0.47	**40**	6	*TG12K10*	0.32
